# Delusions of body control: Psychopathological description of a case.

**DOI:** 10.1192/j.eurpsy.2023.2123

**Published:** 2023-07-19

**Authors:** C. M. Gil Sánchez, J. A. Salomón Martínez, E. Fernández García

**Affiliations:** 1 Psychiatry, Sistema Andaluz de Salud (SAS), ALGECIRAS; 2 Psychiatry, Sistema Andaluz de Salud (SAS), Cádiz, Spain

## Abstract

**Introduction:**

A considerable number of patients with schizophrenia suffer from somatic passivity or delusions of control. So much so, that Schneider considered them as part of the first-rank symptoms.

In these cases, patients can think that feelings, impulses, thoughts, or actions are controlled or imposed by an external force.

**Objectives:**

The objective is to make a psychopathological description of this symptomatology, based on a case report with Anomalous bodily experiences.

**Methods:**

In this study, we describe the case of a patient with disorder of self-experience. We have conducted a systematic review of the descriptions published to date, regarding this case.

**Results:**

We present the case of a 21-year-old patient who had gone to the emergency services three times for somatic pathology (described as dysesthetic and algic sensations in the throat, stomach and testicles).

In the psychopathological exploration, a delusional narrative is observed, as he refers that these sensations are being provoked by external people, with the aim of harming him.

The patient reports that these people are causing an increase in salivation in his salivary glands, for which he spits repeatedly.

He explains that these people can control his organs using an influencing machine, which in this case consists of a microchip implanted at the retroauricular area, from which they give orders and insult him at the same time.

In this case, a good symptom response was achieved with intramuscular Aripiprazole.

**Conclusions:**

In the experiences of passivity, the patient experiences one event as if it were not his, but inserted into his self from the outside.

In the case of somatic passivity, there is a belief that there are external influences acting on the body. In this case, there was probably a kinesthetic hallucination coupled with an experience of passivity.

Similar to other published cases, this patient complained of being controlled and impaired by some form of contemporary technology. Delusions of control are often associated with delusional explanations about how thought or body can be controlled, in this case, through a microchip.

**Disclosure of Interest:**

None Declared

